# Supplementary material for: Opium as a risk factor for early-onset coronary artery disease: Results from the Milano-Iran (MIran) study
Source: PLoS One. 2023 Apr 19;18(4):e0283707. doi: 10.1371/journal.pone.0283707 (PMC10115251; doi:10.1371/journal.pone.0283707)
Supplement: S2 File — (PDF) [file pone.0283707.s002.pdf]

STROBE Statement—checklist of items that should be included in reports of observational studies.

| | Item No. | Recommendation | Page No. | Relevant text from manuscript |
|---|---|---|---|---|
| **Title and abstract** | 1 | (*a*) Indicate the study's design with a commonly used term in the title or the abstract | Pag. 2 | line 31 |
| | | (*b*) Provide in the abstract an informative and balanced summary of what was done and what was found | Pag. 2 | line 34-38 |
| **Introduction** | | | | |
| Background/rationale | 2 | Explain the scientific background and rationale for the investigation being reported | Pag. 3 | |
| Objectives | 3 | State specific objectives, including any prespecified hypotheses | Page 3-4 | line 67-74 |
| **Methods** | | | | |
| Study design | 4 | Present key elements of study design early in the paper | Pag. 5 | Line 78-103 |
| Setting | 5 | Describe the setting, locations, and relevant dates, including periods of recruitment, exposure, follow-up, and data collection | Pag. 5 | |
| Participants | 6 | (*a*) *Cohort study*—Give the eligibility criteria, and the sources and methods of selection of participants. Describe methods of follow-up<br>*Case-control study*—Give the eligibility criteria, and the sources and methods of case ascertainment and control selection. Give the rationale for the choice of cases and controls<br>*Cross-sectional study*—Give the eligibility criteria, and the sources and methods of selection of participants | Pag 5 | |
| | | (*b*) *Cohort study*—For matched studies, give matching criteria and number of exposed and unexposed<br>*Case-control study*—For matched studies, give matching criteria and the number of controls per case | Pag 5 | Line 98 |
| Variables | 7 | Clearly define all outcomes, exposures, predictors, potential confounders, and effect modifiers. Give diagnostic criteria, if applicable | Pag 6-8 | |
| Data sources/ measurement | 8* | For each variable of interest, give sources of data and details of methods of assessment (measurement). Describe comparability of assessment methods if there is more than one group | Pag 6 and 7 | Line 106-134 |
| Bias | 9 | Describe any efforts to address potential sources of bias | Pag 7 and 8 | Line 138-148 |
| Study size | 10 | Explain how the study size was arrived at | Pag 8 | Line 148-151 |

Continued on next page

| | | | | |
|---|---|---|---|---|
| Quantitative variables | 11 | Explain how quantitative variables were handled in the analyses. If applicable, describe which groupings were chosen and why | Pag 7 | Line 142 |
| Statistical methods | 12 | (*a*) Describe all statistical methods, including those used to control for confounding | Pag 7 | Line 138-143 |
| | | (*b*) Describe any methods used to examine subgroups and interactions | Pag 7 and 8 | Line 144-148 |
| | | (*c*) Explain how missing data were addressed | Pag 7 and 10 | Line 143 and 167 |
| | | (*d*) *Cohort study*—If applicable, explain how loss to follow-up was addressed<br>*Case-control study*—If applicable, explain how matching of cases and controls was addressed<br>*Cross-sectional study*—If applicable, describe analytical methods taking account of sampling strategy | Page 5 | Line 98 |
| | | (*e*) Describe any sensitivity analyses | na | |
| **Results** | | | | |
| Participants | 13* | (a) Report numbers of individuals at each stage of study—eg numbers potentially eligible, examined for eligibility, confirmed eligible, included in the study, completing follow-up, and analysed | Pag 9 | Line 157 |
| | | (b) Give reasons for non-participation at each stage | na | |
| | | (c) Consider use of a flow diagram | na | |
| Descriptive data | 14* | (a) Give characteristics of study participants (eg demographic, clinical, social) and information on exposures and potential confounders | Pag 9 | Line 158-163 |
| | | (b) Indicate number of participants with missing data for each variable of interest | Pag 10 | Line 167-169 |
| | | (c) *Cohort study*—Summarise follow-up time (eg, average and total amount) | na | |
| Outcome data | 15* | *Cohort study*—Report numbers of outcome events or summary measures over time | na | |
| | | *Case-control study*—Report numbers in each exposure category, or summary measures of exposure | Pag 10 | Line 166 |
| | | *Cross-sectional study*—Report numbers of outcome events or summary measures | na | |
| Main results | 16 | (*a*) Give unadjusted estimates and, if applicable, confounder-adjusted estimates and their precision (eg, 95% confidence interval). Make clear which confounders were adjusted for and why they were included | Pag 11 and 12 | |
| | | (*b*) Report category boundaries when continuous variables were categorized | na | |
| | | (*c*) If relevant, consider translating estimates of relative risk into absolute risk for a meaningful time period | na | |

Continued on next page

| | | | | |
|---|---|---|---|---|
| Other analyses | 17 | Report other analyses done—eg analyses of subgroups and interactions, and sensitivity analyses | Pag11 and 12 | |
| **Discussion** | | | | |
| Key results | 18 | Summarise key results with reference to study objectives | Pag 13 | Line 210-213 |
| Limitations | 19 | Discuss limitations of the study, taking into account sources of potential bias or imprecision. Discuss both direction and magnitude of any potential bias | Pag 15 and 16 | Line 271-295 |
| Interpretation | 20 | Give a cautious overall interpretation of results considering objectives, limitations, multiplicity of analyses, results from similar studies, and other relevant evidence | Page 15 and 16 | Line 271-295 |
| Generalisability | 21 | Discuss the generalisability (external validity) of the study results | Pag 17 | Line 298-303 |
| **Other information** | | | | |
| Funding | 22 | Give the source of funding and the role of the funders for the present study and, if applicable, for the original study on which the present article is based | Pag 18 | Line 318 |

*Give information separately for cases and controls in case-control studies and, if applicable, for exposed and unexposed groups in cohort and cross-sectional studies.

**Note:** An Explanation and Elaboration article discusses each checklist item and gives methodological background and published examples of transparent reporting. The STROBE checklist is best used in conjunction with this article (freely available on the Web sites of PLoS Medicine at http://www.plosmedicine.org/, Annals of Internal Medicine at http://www.annals.org/, and Epidemiology at http://www.epidem.com/). Information on the STROBE Initiative is available at www.strobe-statement.org.
