# Supplementary material for: Opium as a risk factor for early-onset coronary artery disease: Results from the Milano-Iran (MIran) study
Source: PLoS One. 2023 Apr 19;18(4):e0283707. doi: 10.1371/journal.pone.0283707 (PMC10115251; doi:10.1371/journal.pone.0283707)
Supplement: Attachment — Submitted filename: Opium_MIRAN_PlosOne.docx [file pone.0283707.s003.docx]

**Recreational opium use as a risk factor for coronary artery disease: results from the premature coronary artery disease Milano-Iran (MIran) study**

Alberto Maino, MD, PhD^* †^, Saeed Sadeghian, MD^‡^, Ilaria Mancini, PhD^§^, Seyed Hesameddin Abbasi, MD, PhD^‡ ¶^, Hamidreza Poorhosseini, MD^‡^, Mohammad Ali Boroumand, MD^‡^, Masoumeh Lotfi-Tokaldany, MD, PhD^‡^, Arash Jalali, PhD^‡^, Maria Teresa Pagliari, MSc^*^, Frits Rosendaal, MD, PhD^** ††^, Flora Peyvandi, MD, PhD^* §^

* Fondazione IRCCS Ca’ Granda Ospedale Maggiore Policlinico, Angelo Bianchi Bonomi Hemophilia and Thrombosis Center and Fondazione Luigi Villa, Milan, Italy.

† Azienda Provinciale per i Servizi Sanitari, Ospedale di Tione, Unit of Internal Medicine, Trento, Italy.

‡ Tehran Heart Center, Tehran University of Medical Sciences, Tehran, Iran.

§. Università degli Studi di Milano, Department of Pathophysiology and Transplantation, Milan, Italy.

¶ Bernard Lown Scholar in Cardiovascular Health, Department of Global Health and Population, Harvard T.H. Chan School of Public Health, Boston, MA, USA.

** Leiden University Medical Center, Department of Clinical Epidemiology, Leiden, The Netherlands.

†† Leiden University Medical Center, Department of Thrombosis and Haemostasis, Leiden, The Netherlands.

**Running title:** Opium as a risk factor for coronary artery disease

Character count (title) - 145

Word count (abstract) – 250

Word count (main body of text) – 2904

Number of references - 25

Number of tables – 3

**Correspondence to:**

Saeed Sadeghian, Department of Cardiology, Tehran University of Medical Sciences, Tehran Heart Center, North Kargar Street, Tehran 1411713138, Iran. Tel: +98 21 88029720.‎ Fax: ‎+98 21 8802970

e-mail: [ssadeghian@sina.tums.ac.ir](mailto:ssadeghian@sina.tums.ac.ir)

**Keywords:** Coronary Artery Disease; Opium; Risk Factors; Thrombosis; Young Adult.

**Abstract**

The spreading of recreational opium use pose new health related concerns. In some areas of Asia its use is believed to protect from cardiovascular disorders, such as coronary artery disease (CAD). However, whether opium use has an association with CAD is unclear. We aimed to investigate the association between opium use and CAD. We set up a case-control analysis, i.e., the premature CAD Milano-Iran (MIran) study by enrolling consecutive young patients who underwent a coronary angiography at the Tehran Heart Center, between 2009 and 2012. Incident cases with CAD were contrasted with controls for recreational opium use. Relative risks were calculated in terms of odds ratios (ORs) by logistic regression models adjusted for age, sex, cigarette smoking, body mass index, hypertension, hyperlipidaemia, and diabetes. Interaction analyses were performed between opium and major cardiovascular risk factors. 1011 patients with CAD (mean age 43.6 years) and 2002 controls (mean age 54.3 years) were included in the study. Habitual opium users had a 3.8-fold increased risk of CAD (95%CI 2.4-6.2) compared with non-users. The association was strongest for men, with a fully adjusted OR of 5.5 (95%CI 3.0-9.9). No interaction was observed for the combination of opium addiction and hypertension, or diabetes, but an excess in risk was found in opium users with hyperlipidaemia (OR 16.8, 95%CI 8.9-31.7, expected OR 12.2), suggesting supra-additive interaction. In conclusion, despite common beliefs, we showed that recreational opium use is associated with an increased risk of CAD, even when other cardiovascular risk factors are taken into account.

**Introduction**

Opium is a substance derived from the opium poppy plant, the derivatives of which are used for medical and non-medical purpose.(1) In 2016, according to the last World Drug Report, the opium production worldwide reached the highest estimate ever recorded by the UNODOC (United Nations Office for Drugs and Crimes), driven by a dramatically increased demand for non-medical purpose. There were about 19.4 million opiates users worldwide in 2016, roughly 0.4% of the population aged 15 to 64 years, with more than half of the estimated number of annual opiate users residing in Asia.(2) Apart from being a social problem that could impose persistent and harmful effects on the total population, opium addiction is considered a personal risk.(3) Opium intake and dependence are detrimental to health and can result in injury and poor quality of life.(4) Current evidence suggests that opium consumption is associated with an increased risk of several comorbidities, including cancer.(5,6) Nevertheless, a popular belief, even among some physicians in western and central Asia, is that long-term use of low dose opium has a protective effect against chronic diseases such as diabetes mellitus, hypertension and even atherosclerosis, with the potential to prolong survival by preventing cardiovascular disorders.(7)

Coronary artery disease (CAD) and ischaemic stroke, the most common and severe cardiovascular disorders worldwide, are the leading cause of death in western countries in both men and women. In the last decades a dramatic increase in their incidences in south Asian countries has been observed.(8–10) Despite the common belief and the general awareness about drug abuse, few studies have investigated the association between cardiovascular disorders and opium consumption. Their results are inconclusive for CAD and ischaemic stroke.(11,12) However, there is evidence that opium consumption is associated with an increased risk of death from several causes, including cardiovascular diseases.(13)

With this background, we set up a case-control analysis in the frame of the premature CAD Milano–Iran (MIran) study to investigate the association between recreational opium consumption and the risk of CAD in a young population.

**Patients and Methods**

*Patients*

Young subjects (males <45 years, females <55 years) who underwent diagnostic coronary angiography (CA) at the Tehran Heart Center (Iran) between 2009 and 2012 were screened for the inclusion in the Milano-Iran (MIran) study.(14) The population consists of patients with past or recent history of acute myocardial infarction, stable or unstable angina, atypical chest pain (with positive exercise test or myocardial nuclear scan), valvular heart disease candidate for catheterization, and subjects with peripheral vascular disease (aortic problems, renal artery stenosis, carotid artery stenosis).

Consecutive patients with luminal stenosis greater than 50% in at least one main coronary artery or its branches at the CA were included as cases (severe CAD). Patients with evidence of minimal CAD (luminal stenosis <50%) were excluded from the analysis, as well as patients with history of myocardial infarction.

Controls belongs from the population of the Tehran Cohort Study. The Tehran Cohort Study is a population based cohort that has recently enrolled more than 8000 voluntary participants from the general population of Tehran city with the scope of detecting the risk factors for cardiovascular disease, trauma and their related psychological factors. To be included in the Teheran Cohort Study participants should be more than 35 years old at the time of recruitment. Subjects with no previous history of confirmed CAD, coronary angioplasty, coronary artery bypass surgery, myocardial infarction, symptoms of stable or unstable angina, or known symptomatic valvular heart disease where randomly chosen from the cohort and matched for sex.

The study was approved by the Ethical committee Board of Tehran Heart Center, and all patients and controls signed informed consent before inclusion in the study.

*Clinical variables*

The information regarding demographic and clinical data were retrieved from the patients’ medical records and from a confidential and detailed questionnaire filled by the participants. This included information regarding age, sex, ethnicity, and the presence of cardiovascular risk factors including body mass index (BMI), current or past tobacco use, and history of hypertension, hyperlipidaemia and diabetes. BMI was calculated as weight in kilograms (kg) divided by height in squared meters. Current smokers were those who smoked at least one cigarette per day, or who had stopped smoking for less than one year; former smokers were those who had stopped smoking for at least one year, and never smokers were those who have never smoked. Subjects were considered hypertensive in the presence of a blood pressure repeatedly higher that 140/90 mmHg or the use of antihypertensive medications. Hyperlipidaemia was defined as hypercholesterolemia (total cholesterol greater than 200 mg/dl) or hypertriglyceridemia (triglycerides greater than 200 mg/dl), or history of antihyperlipidemic drugs. Diabetes mellitus was considered in the presence of fasting blood glucose greater than 126 mg/dl or blood glucose greater than 200 mg/dl after oral glucose tolerance test, or in the presence of a history of diabetes mellitus, with or without the use of anti-diabetic medications. Therefore, no distinction was made between different types of diabetes mellitus.

Detailed information on the habits of opium consumption were retrieved. Patients were asked whether they were using or had ever used opium for recreational purpose, whether the use was occasional or habitual, the pattern of assumption (oral or smoked) and the amount of intake in terms of grams per day. For the purpose of the present study, opium addiction was defined as using opium for more than one year habitually on the time of the enrolment or in the past. Therefore, the definition of opium addiction was self-reported in both cases and controls.

*Theory/calculation*

Mean and standard deviation were used to describe continuous variables, count and percentage for categorical ones. Multivariable logistic regression models were used to calculate odds ratios (OR) and corresponding 95% confidence intervals (CI) as measures of relative risk for the association between opium addiction and CAD. In model 1 analyses were adjusted only for age and sex, in model 2 for cigarette smoking, and in model 3 for BMI (included as continuous variable) and history of hypertension, dyslipidaemia and diabetes. The main analysis was stratified by sex, in order to investigate whether sex differences could have a role in the association between opium and CAD. In a secondary analysis, dummy variables were created to assess the combined effect on the risk of CAD of opium addiction and the presence of a major cardiovascular risk factor, such as hypertension, hyperlipidaemia and diabetes. Statistical analyses were performed by using the SPSS statistical software package (IBM SPSS Statistics for Windows, Version 22.0. Armonk, NY: IBM Corp).

**Results**

One thousand and eleven patients with severe CAD and 2002 controls were included in the analysis. Demographic and clinical characteristics are summarized in **Table 1**. Cases were slightly younger than controls (mean age 46.6 vs 54.3), and among cases there was a greater prevalence of women (65.2% among cases and 56.7% among controls). As expected, known cardiovascular risk factors were more prevalent in cases than in controls. The prevalence of opium addiction in cases was 9.3% (94/1011), whereas in controls it was 3.3% (67/2002). Almost all opium addicted subjects consumed opium by smoking.

Opium addiction was associated with a 4-fold increased risk of CAD (OR 4.3, 95% CI 3.0 - 6.2), and this excess in risk decreased only slightly when all the major cardiovascular risk factors were taken into account (fully adjusted OR 3.8, 95% CI 2.4 - 6.2, **Table 2**). When analyses were stratified by sex, male opium users had more than 5-fold increased risk of CAD compared with male opium non-users (fully adjusted OR 5.5, 95% CI 3.0 - 9.9). This estimate was greater than the corresponding one in females (fully adjusted OR 2.6, 95% CI 0.5 - 12.6). However, among females, the overall prevalence of opium addiction was very low (10/1784, 0.6%).

**Table 3** shows the combined effect of opium addiction and the presence of major cardiovascular risk factors. Hypertension was associated with an increased risk of CAD (OR 6.0, 95% CI 4.7 - 7.7). Opium addicted subjects without hypertension had a risk of CAD similar to that of the main analysis (OR 5.0, 95% CI 2.9 - 8.5). The risk of CAD conferred by the combination of opium addiction and hypertension was similar to the one expect by the sum of the two separate relative risks, making the presence of any biological interaction unlikely (OR for the combination 9.0, 95% CI 3.7 - 21.7; expected OR 1 + [6.0 - 1] + [5.0 - 1] = 10, being 1 the baseline risk). A small excess in risk was observed for the combination of opium addiction and hyperlipidaemia. Subjects with hyperlipidaemia had an increased risk of CAD (OR 7.2, 95% CI 5.7 - 9.1), while the relative risk was high in hyperlipidaemic subjects with opium addiction (OR 16.8, 95% CI 8.9 – 31.7; expected OR 1 + [7.2 - 1] + [6.0 - 1] = 12.2), suggesting a positive interaction between opium and high serum lipids levels. On the contrary, diabetic patients with opium addiction had a lower risk of CAD than the sum of the two separate risk factors (OR 1.8, 95% CI 0.5 - 5.9; expected OR 1 + [2.8 - 1] + [5.0 - 1] = 6.8). However, in the latter analysis, numbers of patients with both risk factors were relatively small.

**Discussion**

In a large young population from Iran, we investigated the association between recreational opium consumption and the risk of premature CAD. We found that opium users were at an increased risk of CAD (OR 4.3, 95% CI 3.0 - 6.2) compared with non-users, even when other major cardiovascular risk factors were taken into account.

Very few studies have investigated the relationship between opium addiction and the risk of arterial thrombosis, with small sample size and with contrasting results, ranging from a protective to a harmful effect. Opium has been reported to be protective against the risk of ischaemic stroke, and not affecting the risk of ischaemic hearth disease in a previous study.(11) However, Masoomi et al. suggested that opium use is associated with an increased risk of CAD in cases without cigarette smoking but not in addicted cases with cigarette smoking, by investigating a small population in Kerman, Iran (a case control study including 58 cases and 33 controls).(15) Same conclusion was reported by Sadeghian et al., reporting unadjusted OR for the relationship between opium consumption and CAD in men of 4.5 (95% CI 1.5 - 13.4), very close to the ones found in our study.(16) In a previous study by Khademi et al., opium consumption has been associated with an increased risk of overall death in men and women, for major causes of death, including death from cardiovascular disease, and for different subtypes of opium and various routs of use.(13) The authors discussed whether this association was causal, or confounded by other shared risk factors, primarily the deleterious effects of smoking. Since smoking is the most used route of opium consumption (through smoking the absorption of morphine and codeine, the two sedative alkaloids that bind to the μ opioid receptor (MOR) in the brain, is quick across the lining of the lungs, and they achieve the target tissues within seconds), cigarette smoking is widespread among opium users. However, our results showed that the association between opium consumption and CAD decreased only slightly when smoking was taken into account (OR adjusted for smoking 2.9, 95% CI 2.0 - 4.3), and remained high even when other major cardiovascular risk factors were considered (fully adjusted OR 3.8, 95% CI 2.4 - 6.2). Our results from a large study confirm that opium addiction is a risk factor for CAD and suggest a causal relationship between the increased cardiovascular mortality found by Khademi et al., and opium consumption.

However, the biological mechanisms underlying this association are still to be clarified. Opium and its components might have a direct effect on the vessel wall, affecting atherosclerotic plaque formation and proliferation, according to what has been suggested in a preclinical study on rats.(17) There is paucity of data with regard to the effects of opiates and opium on blood pressure. Acute or chronic exposure to morphine in rats significantly decreased the systolic, diastolic, and mean arterial blood pressures.(18) In humans, however, although opium might temporarily reduce blood pressure by vasodilation, no differences has been found in long term diastolic and systolic blood pressure between opioid users and non-users(19,20), even though an association with chronic kidney disease, which may affect the blood pressure, has been reported.(21) In our analysis, adjustment for hypertension did not change the results. Moreover, we found no interaction between hypertension and opium use in our population. Since the relative risk of CAD for hypertensive subject who used opium was closed to the expected one by the sum of the relative risk of hypertension and opium use, it seems that the two risk factors act on non-interacting pathways. On the contrary, in our study, patients with dyslipidaemia who used opium had an excess risk of CAD compared with dyslipidaemic patients who did not use opium (OR for the combination 16.8, 95% CI 8.9 - 31.7), suggesting a positive biological interaction between high lipids levels and opium. There is little evidence that opium use is associated with increased lipid levels in humans.(20) However, our observation is supported by animal models, in which opium consumption had worsening effects on atherosclerosis formation related with hypercholesterolemia, mainly affecting lipid profile.(17) Finally, the effect of opium on serum glucose levels is controversial. Studies have indicated that opium consumption is related with either an unaltered or a reduced serum glucose levels.(22,23) In our study, diabetic patients who used opium seemed to be at a relatively lower risk of CAD compared with diabetic patients who did not use opium, suggesting a protective effect of opium on the risk of CAD in diabetic patients. However, this analysis included few subjects, and therefore, should be considered very cautiously.

*Strength and limitations*

Our study has several limitations. First, the use of opium was self-reported in our study. The prevalence of recreational opium consumption in the Iranian population has been reported to be as high as 14% in the Golestan cohort, much higher than the prevalence we have found within our controls subjects (3.3%).(24) Part of this difference is related to the case-control design of our study, to the different study populations, and to the definition of opium users, that in our study included only regular users. However, although it has previously been demonstrated in an Iranian population that a self-reported use of opium can be a reliable measurement of its real consumption(25), an underestimation of the real prevalence of opium users in both cases and controls may have occurred. This might partly explain the low prevalence of opium consumption within women, especially when compared with men (0.4% in control women compared with 7.3% in control men). In our analysis, the association between opium and CAD was stronger for men (OR 5.5, 95% CI 3.0 - 9.9) than for women (OR 2.6, 95% CI 0.5 - 12.6). Because the number of exposed women in the latter analyses is too small, we cannot argue that this difference is related to a specific sex effect, or merely to chance. Differential misclassification is less likely to have occurred. Since there is no perception that opium might cause myocardial infarction, it is unlikely that an underestimation of the exposure occurred differently between cases and controls. Second, due to lack of data, we were unable to perform additional analyses on dose related response and route of consumption. Those analyses might have helped in understanding the biological mechanisms underlying the association. Third, although we adjusted the analysis for several factors, we cannot exclude that residual confounding, such as for example physical activity, associated substance abuse, and medications, might have played a role in our results. Finally, despite our study is the largest on the relationship between opium and CAD, numbers in some sub-analyses were relatively small, leading to uncertainty and wide confidence intervals.

**Conclusion**

In conclusion, we found that recreational opium use is associated with an increased risk of CAD, even when several major cardiovascular risk factors are taken into account. The increased risk is consistent in both sexes and it is particularly high for subjects with hyperlipidaemia. Our results should help implementing prevention and educational programs against the non-medical use and spread of opium consumption worldwide, especially in Asian countries.

**Declarations**

*Ethics approval and consent to participate:* The study was approved by the Ethical committee Board of Tehran Heart Center, and all patients and controls signed informed consent before inclusion in the study.

*Conflicts of interest:* The authors declare that they have no conflicts of interest.

*Funding:* The Tehran Heart Center and the Università degli Studi di Milano provided logistical and staff support, without taking part in the design, data collection, analysis, interpretation and manuscript writing.

*Authors' contributions:* A. Maino designed the study, analysed the data, interpreted the results and draft the manuscript. S. Sadeghian collected data, interpreted the results and reviewed the manuscript. I. Mancini analysed the data, interpreted the results and reviewed the manuscript. S. H. Abbasi designed the study, collected data, interpreted the results and reviewed the manuscript. H. Poorhosseini collected data and reviewed the manuscript. M. A. Boroumand collected data and reviewed the manuscript. M. Lotfi-Tokaldany collected data and reviewed the manuscript. A. Jalali collected data and reviewed the manuscript. M. T. Pagliari analysed the data, interpreted the results and reviewed the manuscript. F. Rosendaal designed the study, interpreted the results and reviewed the manuscript. F. Peyvandi designed the study, interpreted the results and reviewed the manuscript. All authors read and approved the final version of the manuscript.

*Availability of data and materials:* supporting data can be available upon request.

**References**

1.	UNODC. World Drug Report 2018 - book 1. World Drug Report 2018. Vienna, Austria: United Nations; United Nations; 2018. 135–152 p.

2.	UNODC. World Drug Report 2018 - book 3. World Drug Report 2018. Vienna, Austria: United Nations; United Nations; 2018. 1–72 p.

3.	Fallahzadeh MA, Salehi A, Naghshvarian M, Fallahzadeh MH, Poustchi H, Sepanlou SG, et al. Epidemiologic study of opium use in pars cohort study: A study of 9000 adults in a rural Southern Area of Iran. Arch Iran Med [Internet]. 2017;20(4):205–10.

4.	Arab M, Kohan M, Amiri M, Mirrashidi SS, Ranjbar H, Arab M, et al. Quality of Life, Social Desirability and Their Relationship in Opium Addicted Persons in Southeast of Iran. Glob J Health Sci. 2014;6(3):97–103.

5.	Kamangar F, Shakeri R, Malekzadeh R, Islami F. Opium use: An emerging risk factor for cancer? Lancet Oncol. 2014;15(2):e69–77.

6.	Ahmadi B, Alimohammadian M, Yaseri M, Majidi A, Boreiri M, Islami F, et al. Multimorbidity: Epidemiology and risk factors in the golestan cohort study, Iran a cross-sectional analysis. Med (United States). 2016;95(7):e2756.

7.	Safaii N, Kazemi B. Effect of opium use on short-term outcome in patients undergoing coronary artery bypass surgery. Gen Thorac Cardiovasc Surg. 2010;58(2):62–7.

8.	Gaziano TA, Bitton A, Anand S, Abrahams-Gessel S, Murphy A. Growing Epidemic of Coronary Heart Disease in Low- and Middle-Income Countries. Curr Probl Cardiol. 2010b;35(2):72–115.

9.	Khoo CM, Tai ES. Trends in the incidence and mortality of coronary heart disease in Asian pacific region - The Singapore experience. J Atheroscler Thromb. 2014;21(SUPPL. 1):2–8.

10.	Hsu J, Brożek JL, Terracciano L, Kreis J, Compalati E, Stein AT, et al. Application of GRADE: making evidence-based recommendations about diagnostic tests in clinical practice guidelines. Implement Sci [Internet]. 2011;6:62.

11.	Rezvani MR, Ghandehari K. Is opium addiction a risk factor for ischemic heart disease and ischemic stroke? J Res Med Sci. 2012;17(10):958–61.

12.	Ebrahimi H, Haghjoo Javanmard S, Asgary S, Dehghani L, Amiri M, Saadatnia M. Opium Addiction and Ischemic Stroke in Isfahan, Iran: A Case-Control Study. Eur Neurol. 2018;79(1–2):82–5.

13.	Khademi H, Malekzadeh R, Pourshams A, Jafari E, Salahi R, Semnani S, et al. Opium use and mortality in Golestan Cohort Study: prospective cohort study of 50 000 adults in Iran. Bmj. 2012;344:e2502–e2502.

14.	Poorhosseini H, Abbasi SH. Cardiac centre of excellence: The Tehran heart center [Internet]. Vol. 39, European Heart Journal. Narnia; 2018. p. 2695–6.

15.	Masoomi M, Arash Ramezani M, Karimzadeh H. The relationship of opium addiction with coronary artery disease. Int J Prev Med. 2010;1(3):182–6.

16.	Sadeghian S, Graili P, Salarifar M, Karimi AA, Darvish S, Abbasi SH. Opium consumption in men and diabetes mellitus in women are the most important risk factors of premature coronary artery disease in Iran. Int J Cardiol. 2010;141(1):116–8.

17.	Mohammadi A, Darabi M, Nasry M, Mohammad-Javad, Saabet-Jahromi, Malek-Pour-Afshar R, et al. Effect of opium addiction on lipid profile and atherosclerosis formation in hypercholesterolemic rabbits. Exp Toxicol Pathol. 2008;61(2):145–9.

18.	Shanazari AAP, Aslani Z, Ramshini E, Alaei H. Acute and chronic effects of morphine on cardiovascular system and the baroreflexes sensitivity during severe increase in blood pressure in rats. ARYA Atheroscler. 2011;7(3):111–7.

19.	Roohafza H, Talaei M, Sadeghi M, Haghani P, Shokouh P, Sarrafzadegan N. Opium decreases the age at myocardial infarction and sudden cardiac death: A long-and short-term outcome evaluation. Arch Iran Med. 2013;16(3):154–60.

20.	Najafipour H, Beik A. The impact of opium consumption on blood glucose, serum lipids and blood pressure, and related mechanisms. Front Physiol. 2016;7:1–13.

21.	Sepanlou SG, Barahimi H, Najafi I, Kamangar F, Poustchi H, Shakeri R, et al. Prevalence and determinants of chronic kidney disease in northeast of Iran: Results of the Golestan cohort study. PLoS One. 2017;12(5):1–14.

22.	Najafi M, Sheikhvatan M. Plausible impact of dietary habits on reduced blood sugar in diabetic opium addicts with coronary artery disease. Int Cardiovasc Res J. 2012;6(3):75–8.

23.	Kouros D, Tahereh H, Mohammadreza A, Minoo MZ. Opium and heroin alter biochemical parameters of human’s serum. Am J Drug Alcohol Abuse. 2010;36(3):135–9.

24.	Nalini M, Oranuba E, Poustchi H, Sepanlou SG, Pourshams A, Khoshnia M, et al. Causes of premature death and their associated risk factors in the Golestan Cohort Study, Iran. BMJ Open. 2018;8(7):e021479.

25.	Abnet CC, Saadatian-Elahi M, Pourshams A, Boffetta P, Feizzadeh A, Brennan P, et al. Reliability and validity of opiate use self-report in a population at high risk for esophageal cancer in Golestan, Iran. Cancer Epidemiol Biomarkers Prev. 2004;13(6):1068–70.

**Tables and Figures**

**Table 1. Clinical characteristics and cardiovascular risk factors of incident cases with coronary artery disease (CAD) and controls.**

|  | **CAD**  **n=1011** | **controls**  **n=2002** |
| --- | --- | --- |
|  |  |  |
| **Age (years, sd)** | 46.6 (5.8) | 54.3 (12.7) |
| **Female (%)** | 659 (65.2) | 1135 (56.7) |
| **Smoking (%)** | 281 (27.8) | 329 (16.4) |
| **Hypertension (%)** | 566 (56.0) | 491 (24.5) |
| **Hyperlipidaemia (%)** | 758 (75.0) | 649 (32.4) |
| **Diabetes (%)** | 367 (36.3) | 336 (16.8) |
| **BMI (kg/m^2^, sd)** | 29.7 (5.1) | 28.0 (5.0) |
| **Opium addiction (%)** | 94 (9.3) | 67 (3.3) |

Smoking includes both former and current cigarettes smokers, data available for 100% of cases and 98.9% of controls; BMI, body mass index, data available for 99.7% of cases and 99.3% of controls.

**Table 2. Risk of CAD by recreational opium addiction**

| **Opium addiction** | **Cases**  **n= 1011** | **Controls n=2002** | **OR^1^ (95%CI)** | **OR^2^ (95%CI)** | **OR^3^ (95%CI)** |
| --- | --- | --- | --- | --- | --- |
| **All** |  |  |  |  |  |
| **no** | 917 (90.7%) | 1935 (96.7%) | ref | ref | ref |
| **yes** | 94 (9.3%) | 67 (3.3%) | 4.3 (3.0 - 6.2) | 2.9 (2.0 - 4.3) | 3.8 (2.4 - 6.2) |
|  |  |  |  |  |  |
| **Male** |  |  |  |  |  |
| **no** | 264 (75%) | 804 (92.7%) | ref | ref | ref |
| **yes** | 88 (25%) | 63 (7.3%) | 6.5 (4.1 - 10.3) | 3.8 (2.3 - 6.2) | 5.5 (3.0 – 9.9) |
| **Female** |  |  |  |  |  |
| **no** | 653 (99.1%) | 1131 (99.6%) | ref | ref | ref |
| **yes** | 6 (0.9%) | 4 (0.4%) | 3.0 (0.8 - 10.9) | 2.5 (0.7 - 9.3) | 2.6 (0.5 - 12.6) |

Odds ratios are obtained by multivariate logistic regression. OR^1^ are adjusted for age and sex; OR^2^ are adjusted for OR^1^ and additionally for smoking; OR^3^ are adjusted for OR^2^ and additionally for the presence of hypertension, dyslipidaemia, diabetes and BMI.

**Table 3. Risk of CAD in relation to the combination of recreational opium addiction and the presence of the major cardiovascular risk factors.**

|  |  | **Cases**  **n= 1011** | **Controls n=2002** | **OR^1^ (95%CI)** | **OR^2^ (95%CI)** |
| --- | --- | --- | --- | --- | --- |
| **Opium addiction** | **Hypertension** |  |  |  |  |
| **no** | **no** | 380 (37.6%) | 1461 (73%) | ref | ref |
| **no** | **yes** | 537 (53.1%) | 474 (23.7%) | 10.4 (8.3 - 13.0) | 6.0 (4.7 - 7.7) |
| **yes** | **no** | 65 (6.4%) | 50 (2.5%) | 4.7 (2.9 - 7.5) | 5.0 (2.9 - 8.5) |
| **yes** | **yes** | 29 (2.9%) | 17 (0.8%) | 11.7 (5.3 - 25.9) | 9.0 (3.7 - 21.7) |
|  |  |  |  |  |  |
| **Opium addiction** | **Hyper-lipidaemia** |  |  |  |  |
| **no** | **no** | 218 (21.6%) | 1309 (65.4%) | ref | Ref |
| **no** | **yes** | 692 (69.1%) | 625 (31.2%) | 11.5 (9.3 - 14.3) | 7.2 (5.7 - 9.1) |
| **yes** | **no** | 43 (2.1%) | 34 (3.4%) | 5.6 (3.1 - 9.9) | 6.0 (3.2 - 11.3) |
| **yes** | **yes** | 60 (5.9%) | 24 (1.2%) | 21.4 (11.7 - 39.1) | 16.8 (8.9 - 31.7) |
| **Opium addiction** | **Diabetes** |  |  |  |  |
| **no** | **no** | 558 (55.2%) | 1613 (80.6%) | ref | ref |
| **no** | **yes** | 358 (35.4%) | 321 (16.0%) | 5.5 (4.4 - 6.8) | 2.8 (2.2 - 3.6) |
| **yes** | **no** | 85 (8.4%) | 52 (2.6%) | 3.7 (2.4 - 5.7) | 5.0 (3.0 - 8.4) |
| **yes** | **yes** | 9 (0.9%) | 15 (0.7%) | 3.9 (1.6 - 10.4) | 1.8 (0.5 - 5.9) |

Odds ratios are obtained by multivariate logistic regression. OR^1^ are adjusted for age, sex and smoking; OR^2^ are adjusted for OR^1^ and BMI, and additionally for hypertension, dyslipidaemia and diabetes when indicated.
