# Supplementary material for: Opium as a risk factor for early-onset coronary artery disease: Results from the Milano-Iran (MIran) study
Source: PLoS One. 2023 Apr 19;18(4):e0283707. doi: 10.1371/journal.pone.0283707 (PMC10115251; doi:10.1371/journal.pone.0283707)
Supplement: Attachment — Submitted filename: comments.docx [file pone.0283707.s004.docx]

I would like to congratulate you on this manuscript. However, you should try to make it better before it could be published

1. Please include the IRB number.
2. Most likely CAG instead of CA, please check the international approach to the write.
3. Authors should improve their style of scientific writeup throughout the manuscript. English language correction is deemed necessary.

Abstract:

Pose-poses

other cardiovascular risk- other cardiovascular risks

Introduction:

non-medical purpose- non-medical purposes

demand for non-medical purpose- demand for non-medical purposes

increase in their incidences- increase in ~~their~~ incidences

Patient:

belongs from- belong to

Clinical variables

data were- data was

and from a confidential- and ~~from~~ a confidential

one year habitually on- one year habitually at

For the purpose of the present study- For ~~the purpose of~~ the present study

Discussion:

various routs of use- various routes of use

Since the relative risk of CAD for hypertensive subject- Since the relative risk of CAD for the hypertensive subjects

Conclusion:

should help implementing- should help implement

A. Introduction:
1. In 2016, according to the last World Drug Report- Any new update on 2021 or 2022 report, if present added this
2. In this line “There were about 19.4 million opiates users worldwide in 66 2016, roughly 0.4% of the population aged 15 to 64 years, with more than half of the 67 estimated number of annual opiate users residing in Asia” compare with latest WHO report of worldwide and check any more current update
3. Give some relationship between homocysteine, Increased plasma fibrinogen, plasminogen activator Inhibitor-1 (PAI-1), and opium and CVD.
4. The number of cardiac patients in Iran and around the world since the introduction and description are both compared in this part.
B. Result:
1. To add how the current sample size was computed and details of the sampling procedure used and also the selection of control details.
2. To describe briefly how to select the variables for computing the logistic regression model.
3. How Confounders were identified?
4. Better to rework the result part.
C. In the discussion part:
1. Add- the cardiovascular impact of opium and potential mechanisms of action, Compare this study with other studies from Europe, the Middle East, and Asia.
2. More work have to do also in the discussion part.

Other comments:

1. Which Opium types are included (e.g: teriak, Sukhteh, and Shireh) all kindly give a slight description of this.
2. Any internal validation study has been performed in this population.
3. Any data for liver function tests that are available, if any, should be compared in the analysis.
4. The age group, BMI, and other relevant group relationships should be classified, if at all possible.
