# Supplementary material for: Opium as a risk factor for early-onset coronary artery disease: Results from the Milano-Iran (MIran) study
Source: PLoS One. 2023 Apr 19;18(4):e0283707. doi: 10.1371/journal.pone.0283707 (PMC10115251; doi:10.1371/journal.pone.0283707)
Supplement: Attachment — Submitted filename: Response to Reviewers_R2_final.docx [file pone.0283707.s006.docx]

**Recreational opium use as a risk factor for coronary artery disease: results from the premature coronary artery disease Milano-Iran (MIran) study**

**Response to Reviewers**

We are glad that the reviewers #1 and #2 are satisfied with the revised version of the manuscript, and we are grateful for the new comments of reviewer #4.

Below we provided our point-by-point response.

**Reviewer #4:**

**- R: This revised version has substantial improvements according to previous comments.**

A: We thank the reviewer for this positive comment.

**- R: Considering that opium, at least in Latin America, is considered a serious drug, in my opinion the term recreational for this kind of drug is dangerous and should be used.**

A: We used the term “recreational” in contrast to “opium for medical purpose”, intended to denote the use of opium taken on an occasional basis for enjoyment. However, we recognized that the concept expressed with the word recreational might have some positive consideration, and therefore, we limited the use of such expression in the manuscript, and we changed it when needed with the more neutral expression “non-medical opium use".

The new title now reads as follow:

*“Opium as a risk factor for early-onset coronary artery disease: results from the Milano-Iran (MIran) study”*

**- R: I also contend the use of the term “premature”, since invasive detection of >50% stenosis does not represent premature disease.**

A: Our definition of coronary artery disease (CAD), a stenosis greater that 50% in one or more main coronary arteries, is a common and widely adopted disease terminology. In the same way, premature CAD is usually defined as CAD occurring in men and women younger than 45 and 55, respectively. However, we realized that these cut-offs vary from 45 to 65 years of age, as evident in different studies. We, therefore, changed our term “premature” with “early-onset CAD” since the latter might be more suggestive of the young age of the participants in the study and might be less misleading. Indeed, early-onset CAD contrasts with late-onset CAD, which is usually defined as CAD experienced in men aged >55 years and women aged >65 years.

We changed the title accordingly (see also our previous answer).

**- R:** **I don’t understand the need for discrimination between logistic regression models 2 and 3. Indeed, regarding the models created, I suggest revision by an expert. Please provide a table with the results from the multivariate analysis using the different models and the HR of each variable within the model.**

A: In our study, almost all opium-addicted subjects consumed opium by smoking (mainly only "teriak" opium). Hence, as expected, the prevalence of cigarette smoking habit was greater in opium users than in non-users. Therefore, we had the intention to show whether smoke (a known risk factor for CAD), was the main confounder in the association between opium and CAD. With this aim, we pre-specified a logistic model (Model #2), adjusted only for matching variables, and cigarette smoking.

This has been clarified in the new version of the manuscript (Statistics paragraph now reads: *“In model 1 analyses were adjusted only for age and sex (matching variable), in model 2 for cigarette smoking (being smoking also the major pattern of opium assumption), and in model 3 for…”*.

As requested, we provide below the complete tables (SPSS output) from the multivariate logistic regression models, with the point estimates (exponential values of the coefficient B [ExpB], representing the Odds Ratios), and corresponding 95% confidence intervals.

**Model #1.** Adjusted for age and sex.

| **Variables in the Equation** | | | | | | | | | |
| --- | --- | --- | --- | --- | --- | --- | --- | --- | --- |
|  | | B | S.E. | Wald | df | Sig. | Exp(B) | 95% C.I.for EXP(B) | |
|  |  |  |  |  |  |  |  | Lower | Upper |
| Step 1^a^ | Opium | 1.457 | .187 | 60.452 | 1 | .000 | 4.293 | 2.974 | 6.199 |
|  | Age | -.080 | .005 | 272.139 | 1 | .000 | .924 | .915 | .932 |
|  | Sex | .792 | .093 | 72.146 | 1 | .000 | 2.209 | 1.840 | 2.652 |
|  | Constant | 1.921 | .253 | 57.755 | 1 | .000 | 6.826 |  |  |

**Model #2.** Adjusted for age, sex and cigarette smoking.

| **Variables in the Equation** | | | | | | | | | |
| --- | --- | --- | --- | --- | --- | --- | --- | --- | --- |
|  | | B | S.E. | Wald | df | Sig. | Exp(B) | 95% C.I.for EXP(B) | |
|  |  |  |  |  |  |  |  | Lower | Upper |
| Step 1^a^ | Opium | 1.061 | .198 | 28.862 | 1 | .000 | 2.891 | 1.962 | 4.258 |
|  | Age | -.078 | .005 | 260.150 | 1 | .000 | .925 | .916 | .933 |
|  | Sex | 1.039 | .104 | 99.877 | 1 | .000 | 2.827 | 2.306 | 3.466 |
|  | Smoke | .816 | .119 | 46.673 | 1 | .000 | 2.262 | 1.790 | 2.858 |
|  | Constant | 1.319 | .271 | 23.651 | 1 | .000 | 3.741 |  |  |
| . | | | | | | | | | |

**Model #3.** Adjusted for age, sex, cigarette smoking , history of hypertension, dyslipidaemia, diabetes and BMI.

| **Variables in the Equation** | | | | | | | | | |
| --- | --- | --- | --- | --- | --- | --- | --- | --- | --- |
|  | | B | S.E. | Wald | df | Sig. | Exp(B) | 95% C.I.for EXP(B) | |
|  |  |  |  |  |  |  |  | Lower | Upper |
| Step 1^a^ | Opium | 1.340 | .244 | 30.049 | 1 | .000 | 3.819 | 2.365 | 6.166 |
|  | Age | -.155 | .008 | 399.980 | 1 | .000 | .857 | .844 | .870 |
|  | Sex | .806 | .130 | 38.288 | 1 | .000 | 2.240 | 1.735 | 2.892 |
|  | Smoke | .963 | .151 | 40.539 | 1 | .000 | 2.620 | 1.948 | 3.525 |
|  | Hypertension | 1.739 | .124 | 198.127 | 1 | .000 | 5.693 | 4.468 | 7.253 |
|  | Diabetes mellitus | .962 | .129 | 55.661 | 1 | .000 | 2.616 | 2.032 | 3.369 |
|  | Hyperlipidemia | 1.916 | .115 | 279.572 | 1 | .000 | 6.797 | 5.429 | 8.509 |
|  | BMI | .031 | .011 | 8.483 | 1 | .004 | 1.031 | 1.010 | 1.053 |
|  | Constant | 2.510 | .454 | 30.610 | 1 | .000 | 12.306 |  |  |
|  | | | | | | | | | |

Regarding the suggestion for an expert revision of the statistical models, we had three authors who have a strong background in statistics and epidemiology (AM and SHA has a PhD in Clinical Epidemiology), and one is a senior epidemiologist (FRR is the head of the Department of Epidemiology at Leiden University Medical Center).

**- R: If you calculated a sample size for the control group of 1000 patients. Why did you include 2002?**

A: We thanks the reviewer for this comment. This has been a typo mistake, since the correct number of controls needed to keep a power of 0.8 is indeed 2000. We corrected the error in the text as follows.

*“Considering a prevalence of opium consumption in the Iranian population of 4%, we estimated that with a sample size of 1000 CAD cases and 2000 controls we were able to detect an OR of 1.7 or higher with 0.95 confidence level and a power of 0.8.”*

**- R: In my opinion, the design of the study is somewhat odd, since you cannot be sure whether the subjects within the control group are free from CAD. Aside from that, why didn’t you match age and sex between groups? P values or confidence intervals should be provided for Table 1.**

A: The controls were screened for the presence of clinically evident CAD by ECG and medical and medication hystory suggesting of CAD (including the standardized Persian version of the Rose angina questionnaire to assess chest pain and ischemic cardiac symptoms in the participants). However, we agree with the reviewer that we cannot exclude that misclassification occurred in the control group. However, a misclassification in the control group leads to diluition of the association. Therefore, even if a misclassification had occurred in the control group, the direction of the observed effect between opium and CAD would not have been affected.

Finalluy, p-values in Table 1 can be misleading since they merely measure a statistically significant difference between the groups, but they say nothing about possible confounders. On the other hand, confounders might not differ in a statistically significant way between groups while affecting the relationship between exposure and disease. We, therefore, prefer to keep p values out of Table 1.

**- R: I believe that given such limitations regarding comparisons between groups, it might have been more appropriate using other design, maybe including only patients with suspected CAD within the MIran cohort and discriminating them according to extension indexes such as Leaman or Duke CAD indexes; or even evaluating opium use among patients with acute vs. stable coronary syndromes.**

A: We thank the reviewer for these suggestions. We are now working on a more detailed description of CAD within the MIran cohort, and we think that these analyses could be evaluated in further studies.

**- R: Please explain why the prevalence of opium consumption was so much higher than expected in the power calculation.**

We expected a prevalence of opium consumption in the general population of Tehran of about 4%, based on a previously reported standardized prevalence of 4.7, 95% CI 4.3-5.0 (in the Kerman cohort, Najafipour H. et al., Iranian Journal of Medical Sciences, 2022). In our control group the prevalence of opium addiction resulted slightly lower that what was expected (at 3.3%). The difference might be related to the different characteristics of the populations. Our controls belong to the inhabitants of Tehran city, and it is well known that the prevalence of opium consumption is higher in rural areas compared with the highly urbanized ones. In order to express data more homogeneously throughout the manuscript, we now stated the expected prevalence of opium consumption in the methods as 4%, instead of 0.04, as follow.

*“Considering a prevalence of opium consumption in the Iranian population of 4%, we estimated that with a sample size of 1000 CAD cases and 2000 controls we were able to detect an OR of 1.7 or higher with 0.95 confidence level and a power of 0.8.”*
